# Stent-based delivery of AAV2 vectors encoding oxidation-resistant apoA1

**DOI:** 10.1038/s41598-022-09524-y

**Published:** 2022-03-31

**Authors:** Bahman Hooshdaran, Benjamin B. Pressly, Ivan S. Alferiev, Jonathan D. Smith, Philip W. Zoltick, Cory M. Tschabrunn, Robert L. Wilensky, Robert C. Gorman, Robert J. Levy, Ilia Fishbein

**Affiliations:** 1 Division of Cardiology, The Children’s Hospital of Philadelphia, 3615 Civic Center Blvd, CHOP, ARC, Room 702 C, Philadelphia, PA 19104 USA; 2 Department of Pediatrics, University of Pennsylvania Perelman School of Medicine, Philadelphia, USA; 3 Department of Cardiovascular and Metabolic Sciences, Cleveland Clinic, Cleveland, USA; 4 Department of Medicine, Division of Cardiovascular Medicine, University of Pennsylvania Perelman School of Medicine, Philadelphia, USA; 5 Department of Surgery, University of Pennsylvania Perelman School of Medicine, Philadelphia, USA

**Keywords:** Interventional cardiology, Restenosis, Gene delivery, Biomaterials, Gene therapy

## Abstract

In-stent restenosis (ISR) complicates revascularization in the coronary and peripheral arteries. Apolipoprotein A1 (apoA1), the principal protein component of HDL possesses inherent anti-atherosclerotic and anti-restenotic properties. These beneficial traits are lost when wild type apoA1(WT) is subjected to oxidative modifications. We investigated whether local delivery of adeno-associated viral (AAV) vectors expressing oxidation-resistant apoA1(4WF) preserves apoA1 functionality. The efflux of ^3^H-cholesterol from macrophages to the media conditioned by endogenously produced apoA1(4WF) was 2.1-fold higher than for apoA1(WT) conditioned media in the presence of hypochlorous acid emulating conditions of oxidative stress. The proliferation of apoA1(WT)- and apoA1(4FW)-transduced rat aortic smooth muscle cells (SMC) was inhibited by 66% ± 10% and 65% ± 11%, respectively, in comparison with non-transduced SMC (p < 0.001). Conversely, the proliferation of apoA1(4FW)-transduced, but not apoA1(WT)-transduced rat blood outgrowth endothelial cells (BOEC) was increased 41% ± 5% (p < 0.001). Both apoA1 transduction conditions similarly inhibited basal and TNFα-induced reactive oxygen species in rat aortic endothelial cells (RAEC) and resulted in the reduced rat monocyte attachment to the TNFα-activated endothelium. AAV2-eGFP vectors immobilized reversibly on stainless steel mesh surfaces through the protein G/anti-AAV2 antibody coupling, efficiently transduced cells in culture modeling stent-based delivery. In vivo studies in normal pigs, deploying AAV2 gene delivery stents (GDS) preloaded with AAV2-eGFP in the coronary arteries demonstrated transduction of the stented arteries. However, implantation of GDS formulated with AAV2-apoA1(4WF) failed to prevent in-stent restenosis in the atherosclerotic vasculature of hypercholesterolemic diabetic pigs. It is concluded that stent delivery of AAV2-4WF while feasible, is not effective for mitigation of restenosis in the presence of severe atherosclerotic disease.

## Introduction

Endovascular stenting is a highly effective intervention for alleviating atherosclerotic narrowing in coronary and peripheral arteries. Recent advances related to refinement of stent materials, design, and sustained elution of anti-proliferative drugs from a stent-based depot, reduced the incidence of secondary obstruction, i.e., in-stent restenosis (ISR) to less than 10% of stent-treated patients^1^. However, due to the wide-spread use of stenting in clinical practice, and higher ISR incidence in certain populations, such as diabetics^2^, the number of patients requiring revascularization of previously stented arterial lesions exceeds 180,000 a year in the USA alone^3^. Furthermore, the progression of native atherosclerosis in the stented arterial segments, i.e., neoatherosclerosis (NA^4^) and late stent thrombosis (LST^5^) caused by incomplete endothelial re-growth and endothelial dysfunction inherent to drug-eluting stent (DES) use, further compromise blood flow through the stented artery. A recent meta-analysis demonstrated a sustained occurrence of major adverse cardiac events after bare metal stent (BMS) and DES implantation at the rate of 2%/year between 1 and 5 years with no evidence of plateau, thus advocating for new approaches to improve long-term outcomes of vascular stenting^6^.

Apolipoprotein A1 (apoA1), the major protein constituent of high-density lipoproteins (HDL), has been previously considered as a potential treatment target for restenosis^7–10^ and as a modulator of atherosclerosis progression^11^. However failure of large clinical trials to moderate CVD-related morbidity and mortality through pharmacologic enhancement of HDL levels^12,13^, led to a change of paradigm, fostering a growing understanding that HDL composition and function, rather than total HDL amount, plays a decisive role in the atheroprotective properties of HDL^14^. Currently, the most reliable metric of the functional aptitude of HDL is their cholesterol efflux activity that assesses the ability of the patient’s HDL to remove cholesterol from macrophages under the standardized in vitro conditions^15^. The negative correlation between the cholesterol efflux and prevalence of coronary artery disease was repeatedly established in prospective^16^ and case-cohort^17^ studies. HDL dysfunction in atherosclerosis is further aggravated by concurrent diabetes in a significant fraction of patients^18^. Furthermore, multivariate analyses demonstrated that impaired cholesterol efflux presents an independent risk factor for developing ISR^19,20^, and NA^20^ after implantation of both BMS and DES.

ApoA1 is subject to oxidative modifications that are responsible for the loss of the athero-protective properties of HDL^21–24^. Specifically, myeloperoxidase (MPO)-mediated conversion of four crucial tryptophan (Trp) residues into oxindolyl alanines (2-OH-Trp) at positions 8, 50, 72, and 108 of human apoA1 was associated with loss of cholesterol efflux from cholesterol-laden mouse macrophages by apoA1^21,23,24^. The abundant presence of these oxindolyl moieties was detected in apoA1 extracted from human aortic atherosclerotic plaques^24^, thus establishing the pathological significance of oxidative Trp modification in human disease. Moreover, a recombinant human apoA1(4WF) in which four vulnerable Trp residues were point-mutated into phenylalanines (Phe) retained its cholesterol efflux activity when exposed to MPO-generated oxidants^24^. Likewise, HDL isolated from human apoA1(4WF) transgenic mice have maintained cholesterol efflux under pro-oxidative conditions, while HDL from wild-type transgenic counterparts, apoA1(WT) failed under conditions of oxidative stress to unload cholesterol from macrophages^21^.

The additional rationale for considering apoA1 as a therapeutic agent to mitigate stenting-triggered vascular pathology beyond the enhancement of cholesterol efflux: are the anti-proliferative activity towards smooth muscle cells (SMC), enhancement of endothelial cell (EC) survival and proliferation, inhibition of reactive oxygen species (ROS) production, mitigation of inflammation and augmentation of NO synthesis and bioavailability^10^. While the superiority of the apoA1(4WF) variant compared with apoA1(WT) in sustaining cholesterol efflux under conditions of oxidative stress was demonstrated^24^, the competence of apoA1(4WF) in mediating these pro-healing effects is currently unknown.

Previous experimental attempts to mitigate the restenotic response with apoA1 have exploited local^7^ and systemic^9^ delivery of human recombinant apoA1 (both wild-type and naturally occurring Milano mutant^25^) or apoA1-mimetic peptides^26^. Given the lack of practical methods for local gene delivery to the vasculature, no prior work has exploited gene therapy approaches to target ISR via sustained production of apoA1 in the arterial wall. While only partially investigated in the setting of vascular interventions, the apoA1 gene transfer with helper-dependent adenoviral vectors^27,28^, and AAV vectors^29,30^ showed much promise for the slowing development of atherosclerosis and even reversal of established atherosclerotic plaques in hypercholesterolemic animal models. AAV vectors are currently in clinical use, and their utility as therapeutics is projected to expand in the future^31^. Our group has published on stent-based delivery of AAV vectors of serotype 2 (AAV2) using reversible vector immobilization on stent struts^32^. The underlying chemical strategy is based on the complexation of metal atoms and their oxides on the stent surface with polyallylamine bisphosphonate bearing latent thiol groups (PABT) with the consecutive attachment of protein G, anti-AAV2 antibody (A20) and AAV2 vector to the stent surface.

We therefore hypothesized that local arterial overexpression of apoA1(4WF) with AAV2 could be of therapeutic value for the treatment of restenosis in the presence of severe atherosclerotic disease. Respectively, the goals of our studies were (1) establishing resistance of the apoA1(4WF) variant expressed by the AAV2 system to oxidation with MPO-derived oxidants; (2) comparing effects of AAV2-encoded apoA1(WT) and apoA1(4WF) on proliferation, migration, and inflammatory activation of SMC and EC; (3) investigating reporter gene expression after deployment of AAV2-eGFP-eluting stents in non-diseased pig arteries, and (4) studying anti-restenotic effectiveness of AAV2-apoA1(4WF) delivery stents in a model of hypercholesterolemic diabetic swine (HDS).

## Results

### Expression of apoA1 (WT and 4WF) following AAV2-mediated transduction in various cell types

**Figure 1:**
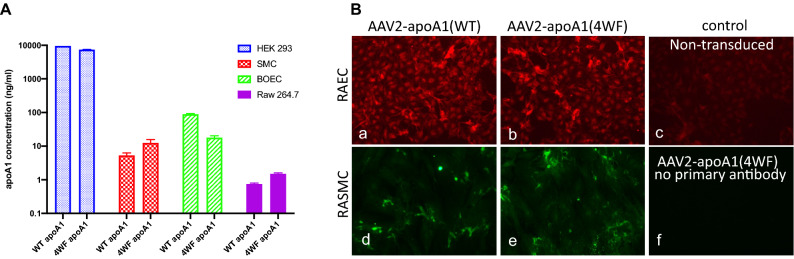
ApoA1 production following AAV2-apoA1(WT) and AAV2-apoA1(4WF) transduction assayed by ELISA and immunofluorescence. (**A**) Human embryonic kidney cells (HEK-293), rat smooth muscle cells (SMC), rat blood outgrowth endothelial cells (BOEC), and murine macrophages (Raw 264.7) were transduced with AAV2-apoA1(WT) and AAV2-apoA1(4WF). ApoA1 production was determined by ELISA of the media conditioned by transduced cells 3 days post-transduction. (**B**) Rat endothelial cells (a-c) and rat smooth muscle cells (d-f) were transduced with either AAV2-apoA1(WT) (a and d) or AAV2-apoA1(4WF) (b, e and f) for 3 days, methanol-fixed and reacted with anti-apoA1 antibody except (c), followed by either Cy 3.5- (a, b and c) or AlexaFluor488-labeled (d, e and f) secondary antibodies. Original magnification is 100×.

To analyze the transduction capacity of AAV2 vectors containing apoA1(WT) and apoA1(4WF) cassettes under control of CMV promoter, Raw 264.7 murine macrophages, rat primary aortic smooth muscle cells (RASMC), human embryonic kidney cells (HEK-293) and rat blood outgrowth endothelial cells (BOEC) were transduced with AAV2-apoA1(WT and 4WF) vectors at multiplicity of infection (MOI) of 10^5^–5 × 10^5^ (10^10^–3.5 × 10^10^ VG/ml). The conditioned media containing lipoprotein free FBS were collected 3 days post-transduction, and the secreted apoA1 variants in the media were determined by ELISA. HEK-293 and BOEC exhibited higher transducibility than SMC, while apoA1 concentrations in the media conditioned by Raw 264.7 macrophages were very low (Fig. 1A). The abundance of WT and 4WF variants in the conditioned media of HEK-293 cells was similar, while in all other cell types, it differed 2–4 fold (WT > 4WF in BOEC, and WT < 4WF in SMC and Raw 264.7). Collectively these results demonstrate that BOEC, and SMC have capacity for AAV2-mediated transduction and apoA1 production. To further confirm the expression of apoA1 variants in the transduced cell lines, rat aortic endothelial cells (RAEC) and RASMC were transduced with AAV2-apoA1 (WT and 4WF) as above and apoA1 (WT and 4WF) expression was confirmed by immunofluorescence. As negative controls, non-transduced RAEC, and RASMC omitted anti-human apoA1 antibody exposure were used. While only light staining was detected in the control groups, AAV2-apoA1(WT)- and AAV2-apoA1(4WF)-transduced cells exhibited distinct cellular staining, confirming apoA1 expression in these cells (Fig. 1B). The staining intensity was higher in RAEC than in RASMC and was comparable between the cells transduced with AAV2-apoA1(WT)- and AAV2-apoA1(4WF) collaborating ELISA results.

### Cholesterol efflux properties of WT and 4WF apoA1 at baseline conditions and in a pro-oxidative environment

**Figure 2:**
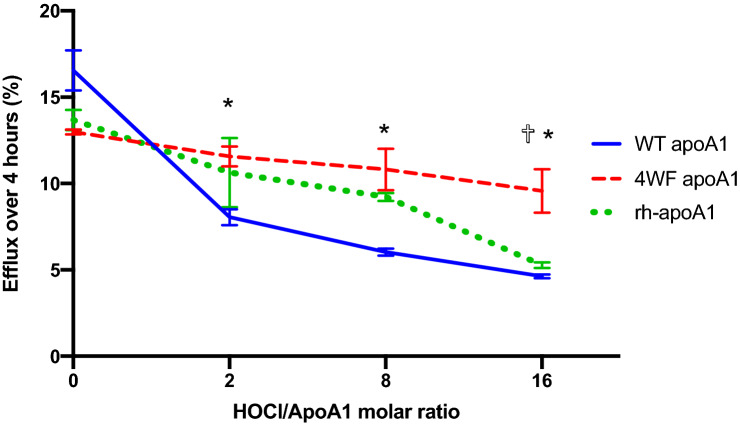
ApoA1(4WF)-, but not apoA1(WT)-mediated efflux following transduction with the respective AAV2 vectors demonstrates oxidative resistance. Cell culture media conditioned by HEK 293 cells transduced with AAV2-apoA1(WT), AAV2-apoA1(4WF), both containing 10 µg/ml of AAV2-encoded apoA1 variants, and the conditioned media from the non-transduced HEK-293 cells supplemented with 10 µg/ml of recombinant human apoA1 (rhapoA1) were incubated with increasing concentrations of HOCl. Cholesterol efflux assays were then performed on ^3^H-cholesterol-loaded Raw 264.7 cells incubated with differently oxidized conditioned media from HEK-293 cells for 4 h. *P < 0.05 4WF vs WT. ✞P < 0.05 4WF vs rhapoA1.

To study cholesterol efflux to the medium conditioned by AAV2-apoA1(WT)- and AAV2-apoA1(4WF)-transduced HEK-293 cells, the collected media were concentrated to 10 µg/ml to match the concentration of recombinant human ApoA1 preparation (rh-apoA1). The media were either used directly in the cholesterol efflux assay or were exposed to escalating concentrations of hypochlorous acid (HOCl) to emulate oxidative stress. The conditioned media from AAV2-apoA1(WT)-transduced cells showed a 27% higher efflux capacity than media from AAV2-apoA1(4WF)-transduced counterparts under basic non-oxidative conditions (HOCl/apoA1 = 0) (Fig. 2). However, rhapoA1 and apoA1(WT) lost 22%, 33% and 59%, and 51%, 64% and 72% of their efflux capacity, respectively at 2, 8 and 16 HOCl/apoA1 molar ratios (Fig. 2). In contrast, the 4WF variant of apoA1 displayed only a 11%, 17% and 26% decrease of its efflux capacity at the same conditions (p < 0.05 for all comparisons between 4WF and both rhapoA1 and WT), therefore indicating oxidative stress resistance of this construct (Fig. 2).

### Effects of AAV2 based apoA1 transduction on the proliferation, migration and inflammatory activation of vascular cell types

**Figure 3:**
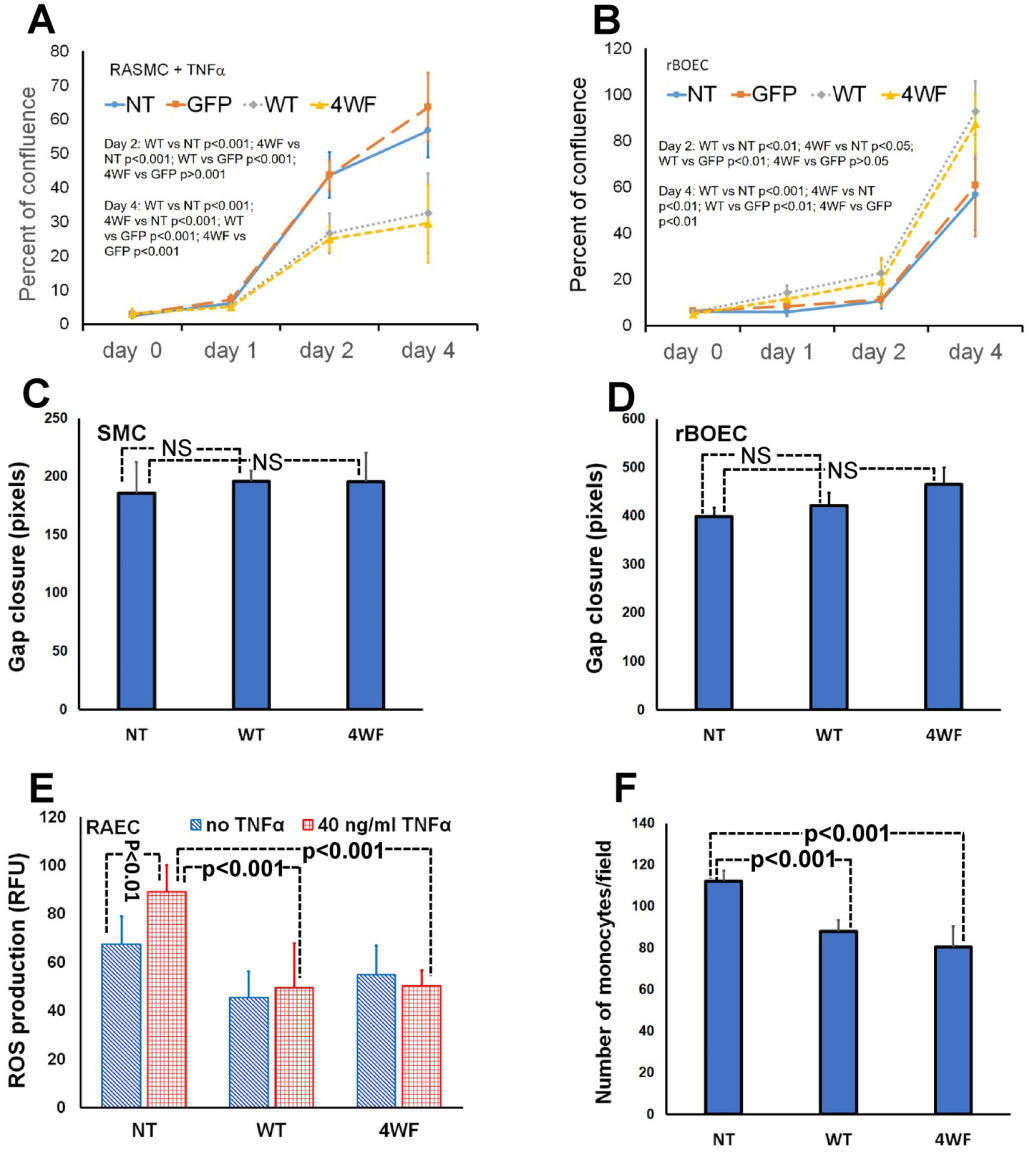
Impact of AAV2-apoA1(WT) and AAV2-apoA1(4WF) transduction on the proliferation and migration of SMC and BOEC, reactive oxygen species (ROS) production, and anti-inflammatory responses in endothelial cells. WST-8 assay was used to evaluate the effect of apoA1(WT) and apoA1(4WF) overexpression on proliferation of (**A**) TNFα (20 ng/ml)-stimulated rat aortic SMC and (**B**) non-stimulated rat BOEC. The results (both **A** and **B**) were normalized by WST-8 assay values produced in confluent cultures of rat SMC (**A**) and rat BOEC (**B**) and expressed as the percent of a monolayer confluency. A monolayer scratch injury assay was used to assess migratory capacity of apoA1(WT)- and apoA1(4WF)-transduced rat SMC (**C**) and rat BOEC (**D**) compared with non-transduced counterparts. A closure of the gap inflicted by pipette tip was quantified 24 h after the scratch injury. (**E**) CM-H2DCFDA assay in non-transduced, apoA1(WT)- and apoA1(4WF)-transduced RAEC with/without TNFα stimulation. (**F**) Attachment of PKH26-labeled rat monocytes to TNFα-stimulated RAEC.

To evaluate the effects of endogenous apoA1 production on the proliferation of transduced cells, RASMC and rat BOEC grown to 70–80% confluence in T-75 flasks were transduced at MOI 10^5^ (10^10^ VG/ml) with AAV2-eGFP, AAV2-apoA1(WT), AAV2-apoA1(4WF) or left untransduced. Four days after transduction, the cells were trypsinized and collected by centrifugation. 5 × 10^3^ non-transduced (NT), AAV2-eGFP-, AAV2-apoA1(WT)- or AAV2-apoA1(4WF)-transduced SMC (Fig. 3 A) and BOEC (Fig. 3B) were seeded on 96-well plates. RASMC were cultured in the presence of 20 ng/ml TNFα to mimic stimulating effects of cytokines present in an atherosclerotic environment on smooth muscle cell proliferation. WST-8 assay was used to measure relative cell numbers at 2 and 4 days post-seeding. In a preliminary experiment, tight correlation (r^2^ = 0.925) between WST-8 generated data and manual cell counts was observed (Supplemental Fig. 1). Compared to NT SMC, the proliferation of SMC transduced with apoA1(WT), and apoA1(4WF) were inhibited by 39% and 43% at day 2, and by 43% and 48% at day 4, respectively (Fig. 3A; p < 0.001 for all comparisons with NT RASMC). GFP transduction did not affect growth of RASMC at day 2 and resulted in statistically insignificant 12% increase compared to NT RASMC at day 4 (Fig. 3A). A significantly faster growth rate was observed in AAV2-apoA1(WT) and AAV2-apoA1(4WF)-transduced BOEC, in comparison with NT counterparts on day 2 (112% and 80%) and day 4 (63% and 54% increase) respectively (Fig. 3B). eGFP transduction did not affect the growth rate of BOEC (Fig. 3B). The growth kinetics of differently transduced RASMC and rat BOEC was further confirmed by Ki-67 immunostaining at day 4 post-seeding, showing a reduced incidence of Ki-67^+^ nuclei in apoA1(WT)- and apoA1(4WF)-transduced RASMC and augmented Ki67 positivity in apoA1(WT)- and apoA1(4WF)-transduced rat BOEC cultures (Supplemental Fig. 2).

Migration of SMC (Fig. 3C) and BOEC (Fig. 3D) assessed by the rate of cellular monolayer restoration after a linear scratch injury was not significantly affected by AAV2-apoA1(WT) and AAV2-apoA1(4WF) transduction.

Non-transduced rat aortic endothelial cells (RAEC) or RAEC transduced with AAV2-apoA1(WT) and AAV2-apoA1(4WF) vectors for 3 days were stimulated with 40 ng/ml rat TNFα for 24 h or were left unstimulated followed by ROS assay with CM-H2DCFDA. ApoA1(WT) and (4WF)-transduced RAEC demonstrated 28% and 22% lower basal levels of ROS production than non-transduced (NT) RAEC. Moreover, while TNFα increased ROS production in NT RAEC (p < 0.005), apoA1(WT)- and apoA1(4WF)-transduced RAEC exhibited resistance to TNFα, resulting in a 45% reduction of ROS production by apoA1(WT) and apoA1(4WF)-transduced TNFα-stimulated RAEC, compared to TNFα-stimulated NT RAEC (p < 0.001) (Fig. 3E). In a separate experiment, the oxidative stress indicator, CellROX was used to determine basal ROS production in differently transduced non-stimulated RAEC, and in RAEC induced with tert-butyl hydroperoxide (tBHP). While basal ROS production was reduced by 12–15% in apoA1-transduced compared to NT RAEC, an increase of ROS production upon tBHP stimulation was dampened 32% in apoA1(4WF) transduced compared to NT RAEC (Supplemental Fig. 3).

To evaluate the effects of apoA1 transduction on inflammatory activation of endothelium, rat monocytes isolated from peripheral blood were fluorescently tagged with PKH-26 dye. 5 × 10^4^ monocytes were then added to the wells with TNFα-stimulated non-transduced RAEC (NT) or RAEC transduced with AAV2-apoA1(WT) and AAV2-apoA1(4WF). Monocytes incubated with NT BOEC exhibited significantly higher attachment when compared to BOEC transduced with WT and 4WF (22% and 33%, respectively; p < 0.001 for both comparisons) (Fig. 3F). Inhibition of monocyte attachment to AAV2-apoA1(WT)—and AAV2-apoA1(4WF)-transduced RAEC monolayers was not caused by AAV2 transduction-related cell signaling since eGFP-transduced RAEC demonstrated a trend to higher monocyte binding (p = 0.067) than NT RAEC (Supplemental Fig. 4).

**Figure 4:**
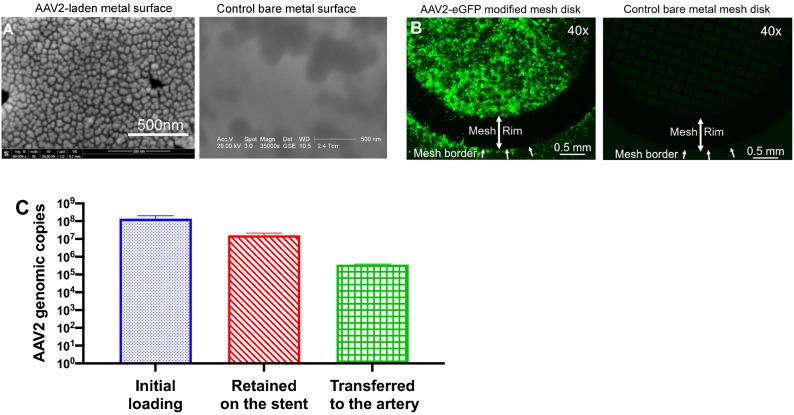
Polybisphosphonate/protein G/antibody-based affinity immobilization of AAV2 particles on stainless steel meshes and cobalt/chromium stents with subsequent vector release and cell transduction. (**A**) Scanning electron microscopy images of AAV2 particles immobilized on the surface of a stainless steel mesh disk (left panel) vs control bare metal disk (right panel). (**B**) Underlying HEK-293 cells were transduced by placement of the mesh disk formulated with AAV2-eGFP (left panel) compared to non-transduced cells treated with the control bare metal mesh disk (right panel). (**C**) Immobilized AAV2 particles were disengaged from the not deployed stents, from the stents deployed in the pig coronary arteries and extracted from the arterial tissue underlying deployed stents. The viral DNA was isolated using a QIAamp DNA minikit and amplified in RT-PCR reaction with AAV2-specific primers. The number of AAV2 genomic copies was quantified using a calibration curve constructed with a known amount of AAV2 genomes.

### AAV2 reporter studies

#### Immobilization of AAV2 vectors on metal surfaces

To characterize our AAV2 delivery system, we first confirmed the immobilization of AAV2 vectors on stainless steel mesh disks. Scanning electron microscopy showed uniform coverage of the surface with the spherical objects of 20–30 nm diameter matching the size and general morphology of AAV2 vectors (Fig. 4A; left panel). These objects are absent on the bare steel surface (Fig. 4A; right panel). Labile tethering of the AAV2 particles with the antibody allows for the timely release of transduction-competent vector particles from the metal substrate and subsequent transduction of proximate cells in vitro. To assure the transduction efficiency of underlying cells with substrate-immobilized AAV2 vectors, circular steel mesh disks were formulated with AAV2-eGFP vectors using the PABT/protein G/anti-AAV2 antibody tethering. Three days after placing the meshes on top of the HEK-293 monolayer in culture, significant GFP expression ensued mainly localized to the area under the mesh (Fig. 4B).

#### Acute in vivo studies-AAV2 stent loading

RT-PCR with AAV2-specific primers was used to determine the number of viral genomes associated with the surface of undeployed stents, stents retrieved from the coronary arteries of the experimental animal 1 h after implantation, and the arterial tissue directly contacting the explanted stents. 1.38 × 10^8^ ± 3.71 × 10^7^ of AAV2 genomes were found in association with the control stents that were not deployed in the animals. The stents explanted at 1 h post-deployment displayed 1.59 × 10^7^ ± 3.1 × 10^6^ viral genomes (i.e., approximately 12% of the initial loading). Finally, 3.65 × 10^5^ ± 1.97 × 10^4^ AAV2 genomes (0.26% of the total vector dose) were transferred to the arterial wall (Fig. 4C).

#### In vivo reporter studies

**Figure 5:**
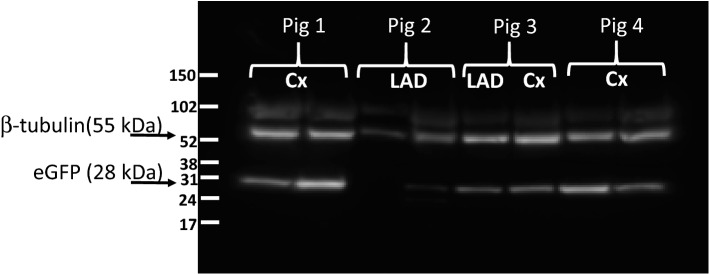
eGFP expression in non-diseased pig arteries treated with AAV2-eGFP stents. Western blot of protein extracts from coronary arteries receiving AAV2-eGFP stents (4 animals, 8 stents). The animals were euthanized 7 days after stent deployment. The extracted proteins from the stented segments were resolved by PAGE, transferred to a nitrocellulose membrane, and probed with anti-eGFP and anti-β-tubulin (loading control) antibodies.

Arterial segments opposing the implanted AAV2-eGFP stents were harvested 7 days after stent deployment in the coronary arteries of healthy pigs and immediately processed for Western blot studies. Western blot analysis of AAV2-eGFP GDS-treated arteries (Fig. 5 and Supplemental Fig. 5) showed the presence of two distinct bands corresponding to molecular weights of eGFP (27 kDa) and β-tubulin (55 kDa) used as a loading control. When normalized to β-tubulin, the relative expression of eGFP varied 4.6-fold across 8 analyzed samples (Supplemental Fig. 5B).

### Hypercholesterolemic diabetic swine studies

#### The prevalence of neutralizing anti-AAV2 antibodies in the plasma of study animals

**Figure 6:**
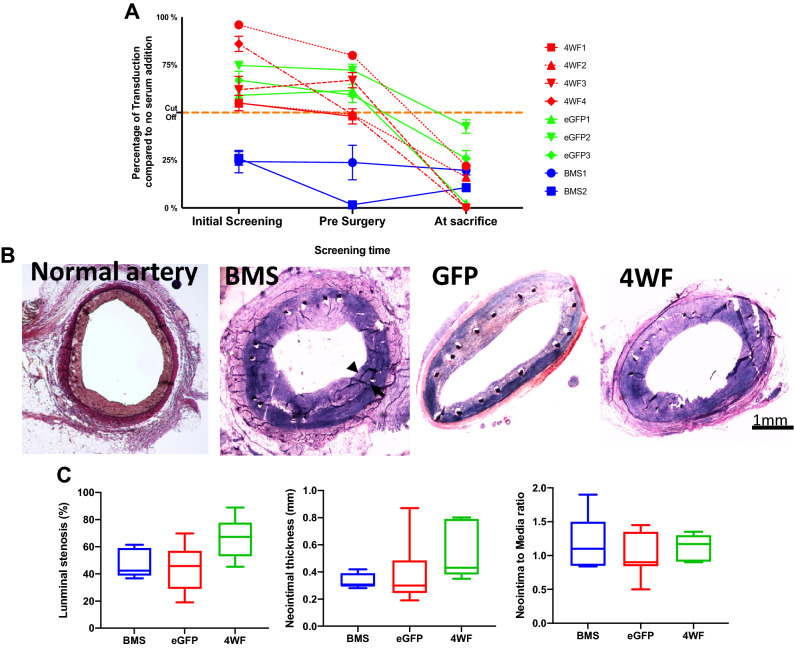
Long-term expression of the therapeutic transgene, the anti-AAV2 neutralizing antibodies dynamics, and the morphometric results in hypercholesterolemic diabetic pigs. (**A**) The percent of retained AAV2eGFP transduction capacity after incubation with 1:20 diluted serum from experimental pigs at different time points throughout the study. NAB positivity was denoted at 50% reduction of transducibility of sera-incubated compared to the not incubated AAV2 vectors. (**B**) Representative images of Verhoeff-van Gieson – stained sections of a normal pig coronary artery and pig coronary arteries treated with BMS, AAV2-eGFP- and AAV2-apoA1(4WF)-eluting stents. (**C**) Morphometric measurements of the stented segments using 3 different calculation methods (percent of luminal stenosis, neointimal thickness, and the ratio of the neointimal area to the medial area).

Blood was collected for measuring titers of AAV2-neutralizing antibodies at the inception of the study, 1 week prior to surgical intervention and at euthanasia. Four out of 13 animals tested positive for the preformed anti-AAV2 antibodies at the beginning of the study. These animals were, therefore, either removed from the study or assigned to the BMS group (Supplemental Fig. 6). Antibody titers either remained unchanged or increased insignificantly after the 24-week period of model development (Fig. 6A). In contrast, plasma of all 9 assayed animals harvested when sacrificed exhibited high levels of neutralizing antibodies exceeding the cut-off values for the assay positivity (more than 50% decrease of transduction at 1:20 plasma dilution compared to the reference control of no plasma supplementation) (Fig. 6A).

#### Effects of stent-based delivery of AAV2-apoA1(4WF) on the formation of restenotic lesions in the coronary arteries of HD pigs

Nine of 11 animals treated with stent angioplasty survived to the 4-week experimental endpoint. The explanted stented arteries were histologically processed. The digital images of Verhoeff-van Gieson—stained sections were acquired (Fig. 6B) and analyzed with ImageJ software. None of the three quantitative indices of restenosis (percent of luminal stenosis, neointima to media ratio, and neointimal thickness) differed significantly (p > 0.05) between the AAV2-apoA1(4WF) group and two controls (BMS, and AAV2-eGFP stents) (Fig. 6C).

#### Human apo1(4WF) expression after implantation of AAV2-apoA1(4FW)-eluting GDS

Expression of human apoA1(4WF) isoform in the porcine arterial tissue located within 2–3 mm of the implanted stents was found in 5 out of 12 analyzed AAV2-apoA1(4FW)-eluting GDS. Low expression levels were exceeded baseline 2.6–8-fold (Supplemental Fig. 7).

## Discussion

The studies reported herein established that AAV-mediated gene therapy with human apoA1(4WF) transgene is capable of rescuing the attenuated efflux of cholesterol from macrophages under physiologically-relevant conditions of oxidative stress. Furthermore, site-directed mutagenesis resulting in four Trp-Phe substitutions in the apoA1(4WF) isoform does not compromise the inherent apoA1 ability to inhibit proliferation of SMC, promote proliferation of endothelial cell lineage, and reduce inflammatory activation of the endothelium. Vascular tissue transduction by AAV2 vectors immobilized on the stent struts was shown to result in short-term expression of the delivered transgene. However, prolonged transduction is compromised by low initial transduction of vascular tissue, pre-existing and, conceivably, by newly induced anti-AAV2 neutralizing antibodies, undermining ultimate therapeutic effects. Further refinements of apoA1(4WF) GDS are needed for potential clinical use.

### The potential role of augmenting apoA1/HDL quantity and functionality in the prevention of in-stent restenosis, late stent thrombosis, and neoatherosclerosis

Re-occlusion of stented arteries with neointimal tissue (ISR), a thrombus (LST), or unremitted expansion of atherosclerotic plaque in the culprit stented lesions (NA) remains the central unresolved problem of interventional cardiology. One of the main limitations of the DES devices is their indiscriminate growth-inhibiting effect towards both SMC and endothelium, resulting in the escalation of thrombotic complications. ApoA1 and HDL (as the physiological carrier of apoA1) are unique in their opposing actions on SMC and endothelium. Previous studies demonstrated that both apoA1 and reconstituted HDL, in physiological concentrations, decrease proliferation of TNFα-stimulated human umbilical SMC^33^ while increasing survival and proliferation of endothelial cells^34^ and endothelial precursors^35^. Likewise, apoA1 and HDL were shown to promote endothelial cell migration *in vitro*^36^, support the restoration of the integral endothelial monolayer in the mouse model^36^, and inhibit SMC migration^37^, thus limiting the number of media-originated SMC in the intimal space.

In addition to the modulation of proliferative and migratory characteristics of SMC and endothelial cells, the beneficial, pro-healing effects of apoA1/HDL in the stented arteries include: increased efflux of cholesterol from foam cells^9^, enhancement of nitric oxide production and bioavailability^38^, mitigation of reactive oxygen species damage^39^, anti-thrombotic activity^40^ and anti-inflammatory effects^41^. Collectively, these traits make apoA1 an appealing choice for the prevention and treatment of vascular pathology precipitated by stenting^10,42^.

### Gene delivery stents

As autonomous implantable devices, DES possess a limited loading capacity that is often inadequate to provide therapeutic concentrations of drug for the entire period of post-angioplasty vessel remodeling. Conversely, stents furnished with gene delivery vectors immobilized on the surface of the struts, i.e., gene delivery stents (GDS), may overcome this intrinsic DES deficiency by delivering the gene vector into the stented vessel wall and making the vascular tissue a permanent production site of the encoded therapeutic protein^43^. We^44^ and others^45^ showed sustained transgene expression in mammalian vasculature upon transduction with AAV2 and AAV5 and the anti-restenotic effectiveness of GDS formulated with inducible nitric oxide synthase-expressing AAV2 in the rat model of carotid stenting^32^. Furthermore, AAV-based expression systems were successfully used to upregulate apoA1 production in vivo upon systemic vector delivery^30^.

### Advantages of apoA1(4WF) over apoA1(WT) in cholesterol efflux under conditions of oxidative stress

Efficient cholesterol efflux in stented patients is associated with a reduced need for reintervention related to ISR^19^ and NA^20^. Platelet aggregation, which underlies LST on the incompletely endothelialized stent surface, is also reversely correlated with cholesterol efflux values^46^. Cholesterol-mobilizing properties of apoA1 are severely impaired by oxidative modification of several key Trp residues^23^. To this end, the oxidation-resistant 4WF apoA1 mutant, in which vulnerable Trp were substituted for Phe, exhibited greater cholesterol efflux than a WT protein in a pro-oxidative environment^21,24^. In our studies, apoA1(4WF) endogenously produced in AAV2-apoA1(4WF) transduced cells was capable of maintaining cholesterol efflux at the most severe conditions of oxidative stress, while the endogenously produced, or exogenously supplemented, WT apoA1 demonstrated a significant reduction of the efflux activity compared to the non-oxidative environment (Fig. 2). It is noteworthy that previously demonstrated oxidation resistance of apoA1(4WF) produced in the prokaryotic expression system^24^, would not necessarily warrant the effectiveness of the apoA1(4WF) produced in the eukaryotic cells, since post-translational modifications affect apoA1 functionality^47^.

### Comparison of apoA1(WT) and apoA1(4WF) as modulators of physiological responses in SMC, EC and BOEC

It is not clear whether the differing performances of WT and 4WF apoA1 variants in the cholesterol efflux assay are mechanistically related to the apoA1 effects on the proliferation and migration of SMC and BOEC and the anti-inflammatory properties of apoA1. We therefore investigated the competence of apoA1(4WF) to exhibit beneficial physiological effects on the primary cell types involved in the pathogenesis of stenting-related complications. We showed a profoundly decreased growth rate of the primary vascular SMC transduced with both AAV2-apoA1(WT) and AAV2-apoA1(4WF) compared to the non-transduced SMC (Fig. 3A), with no significant difference between the two forms of apoA1, and an increased proliferation in AAV2-apoA1(4WF)-transduced BOEC cultures compared to their non-transduced counterparts (Fig. 3B). Since cell quantification assays reflect the combined effects of proliferation and apoptosis on cell growth, the WST-8 results were further corroborated with Ki67 immunostaining which is exclusively associated with cell proliferation (Supplemental Fig. 2). Together, these results in AAV-transduced cells confirm the previously observed modulation of SMC^33^ and EC^34^ proliferative activity with exogenously supplemented apoA1(WT) and indicate a non-inferiority of apoA1(4WF) compared to apoA1(WT) as a therapeutic agent capable of simultaneous inhibition of SMC proliferation and stimulation of EC re-growth.

In contrast to published data^36,37^, we were unable to find any significant impact of either apoA1(WT) or apoA1(4WF) on the migration of SMC and BOEC (Fig. 3C, D). In addition to the different contexts of apoA1 presence in the cell culture (AAV2-driven endogenous expression versus direct exogenous supplementation), this discrepancy may be related to the different methodology^37^ used to measure cell migration.

We also demonstrated the attenuated intracellular ROS production, both under basic conditions and after TNFα stimulation, in AAV2-apoA1(WT)- and AAV2-apoA1(4WF)-transduced EC compared to the non-transduced control (Fig. 3E). ROS mitigation in the apoA1-expressing cells could be contributing to the decreased inflammatory activation of EC upon TNFα stimulation, as evidenced by reduced attachment of fluorescently-labeled syngeneic monocytes to the confluent EC monolayers in vitro (Fig. 3F). These apoA1 activities were previously demonstrated only after supplementing apoA1^41^, or its peptide analog^39^, but never following endogenous production of apoA1 in transduced cells.

### Reporter and therapeutic studies in porcine models

Transduction of porcine peripheral arteries following local intravascular delivery of AAV serotypes 2 and 9 was shown before^48,49^, however to the best of our knowledge, no prior study in pigs has investigated arterial gene transfer by the stent-immobilized AAV vector. To characterize gene transduction of the arterial wall with GDS in the pig model, we used the implantation of AAV2-eGFP eluting stents in the coronary arteries of healthy young animals. eGFP expression was detected in all arteries explanted 7 days after deployment of GDS (Fig. 5 A), albeit in different amounts (Supplemental Fig. 5B).

In a limited set of therapeutic studies comparing the anti-restenotic effectiveness of AAV2-apoA1(4WF) stents with AAV2-eGFP and BMS controls, we were unable to find a superior performance of AAV2-apoA1(4WF)-eluting GDS (Fig. 6 B and C). Analysis of the reasons for this therapeutic failure led us to the detection of high anti-AAV2 antibody neutralizing activity in our experimental animals (Fig. 6 A), even before the iatrogenic AAV2 exposure. At the conclusion of the experiments, at 1 month after deployment of the AAV2-eluting GDS, all animals exhibited the AAV2-neutralizing antibody titers that exceeded the widely accepted^50^ cut-off of 50% reporter transgene activity inhibition in vitro. In a previous study, 30% of pigs were found seropositive for AAV2-specific neutralizing antibodies^51^. This number is close to 40% observed in the current experimental series. While AAV seroprevalence in the human population^52^ clearly presents a formidable hurdle for the realization of the therapeutic potential of AAV2-carrying GDS, several recent reports suggest potential approaches for mitigating the detrimental antibody responses using preemptive systemic administration of empty “decoy” AAV vectors^53^ or preemptive plasmapheresis with AAV antibodies-depleting column^54^. Furthermore, the use of AAV2 vector pseudotyped with serotype 9 capsid proteins (AAV2/9) may be advantageous due to less prevalent preformed neutralizing antibodies^55^ and higher transgene expression^44^ compared with AAV2. In addition to the high titers of neutralizing antibodies, suboptimal transduction of the vasculature with AAV2 vector even in the absence of AAV2-neutralizing activity in the plasma is a major reason for the failure to demonstrate therapeutic effects of GDS. Additionally, attenuation of the CMV promoter activity^56^ may contribute to the low levels of apoA1(4WF) detected in the vascular tissue adjacent to the deployed AAV2-eluting GDS (Supplemental Fig. 7) and the lack of the anti-restenotic effects (Fig. 6 B and C). To this end, substituting the CMV promoter with the native human apoA1 promoter reinforced with four apoE enhancers was previously shown to sustain therapeutic levels of apoA1 expression for more than 6 months in vivo^57^.

### Limitations

There are several significant limitations of the studies reported here. First, while the cholesterol efflux studies showed the superiority of apoA1(4WF) over apoA1(WT) under simulated conditions of oxidative stress, the experimental design chosen for the proliferation, migration, and inflammation assays used a non-oxidative environment, thus establishing non-inferiority rather than the superiority of apoA1(4WF). Second, no neutralizing antibody assays were pursued in the reporter pig studies. This reduces scientific rigor from the interpretation of the mismatch between successful reporter expression at 7 days and the lack of anti-restenotic effects at 28 days post-stenting as a consequence of escalating production of AAV2 neutralizing antibodies in the experimental animals. Third, since no therapeutic benefit of apoA1(4WF)-eluting stents was demonstrated in our studies, the anti-restenotic effectiveness of AAV2-apoA1(WT)-counterparts was not investigated in the animal model. These issues are the subject of our ongoing and planned work.

## Conclusions

These studies demonstrated that AAV2-mediated apoA1(WT) and apoA1(4WF) transduction of the cell types relevant for the pathogenesis of ISR selectively modifies the physiology of SMC and EC, promoting anti-restenotic responses. Cholesterol efflux from the foam cells, hypothetically a crucial process in ISR and NA prevention, is better sustained with apoA1(4WF) than apoA1(WT) over-expression under conditions of MPO-triggered oxidative damage to apoA1 which is prevalent in CVD patients^23^. While GDS formulated with AAV2-eGFP attain transgene expression in the underlying healthy porcine arterial tissue 7 days after stent implantation, AAV2-apoA1(WT) stents implanted in the atherosclerotic arteries of the HDS model did not decrease the severity of ISR, presumably due to the intrinsically low AAV2-mediated transduction of atherosclerotic arteries and the development of dominant humoral immune response against the gene vector. Nevertheless, the prominent anti-atherosclerotic and anti-restenotic properties of apoA1(4WF) warrant further investigations of its potential as a therapeutic moiety in conjunction with gene delivery stents.

## Materials and methods

### Materials, chemicals, and biologicals

316L grade stainless steel foil and mesh disks were from Goodfellow (Coraopolis, PA, USA) and EMS (Hatfield, PA, USA), respectively. Clinical grade L605 cobalt-chromium alloy stents of open cell design were mounted on 3 mm and 3.5 mm balloon catheters. Polyallylamine bisphosphonate with latent thiol groups (PABT) and branched polyethyleneimine with installed pyridyldithio groups PEI(PDT) were synthesized in our laboratory as described before^58^. Tris(2-carboxyethyl)phosphine hydrochloride (TCEP), 8Br-cAMP, CM-H2DCFDA, and CellROX Orange were purchased from Thermo Fisher Scientific (Waltham, MA, USA). Thiolated protein G was acquired from Protein Mods (Madison, WI, USA). Anti-AAV2 (clone A20) and anti-β tubulin antibody were were from GeneTex (Irvine, CA, USA). Anti-human apoA1 antibody was from Abcam (Cambridge, MA, USA). Anti-eGFP antibody was from Rockland Immunochemicals (Limerick, PA, USA). Anti-Ki67 antibody was from Novus Biologicals (Centennial, CO). The secondary AlexaFluor 488 and AlexaFluor 555-labelled antibodies were from Invitrogen (Waltham, MA, USA). Human recombinant apoA1 and *tert*-butyl hydroperoxide (tBHP) were from Sigma-Aldrich (St. Louis, MO, USA). AAV2-human apoA1(WT), AAV2-human apoA1(4WF), and AAV2-eGFP were manufactured by the University of Pennsylvania Vector Core using the triple plasmid transfection protocol^59^. Human apoA1 ELISA assay was purchased from R&D Systems (Minneapolis, MN, USA). WST-8 assay kit was purchased from Cayman Chemical, Ann Arbor, MI, USA). ^3^H-cholesterol was obtained from Perkin Elmer (Waltham, MA, USA). Primary rat aortic smooth muscle (RASMC) and endothelial cells (RAEC) were from Lonza (Rochester, NY, USA) and Gelantis (San Diego), respectively. Human embryonic kidney (HEK-293) and Raw 264.7 macrophage cell lines were from ATCC (Manassas, VA, USA). Rat blood outgrowth endothelial cells (BOEC) were isolated and characterized as described^60^.

### Vector immobilization on metal substrates

A protocol for reversible immobilization of AAV2 vectors to the bare metal surfaces was previously published by our group^32^. Briefly, cobalt/chromium stents and stainless steel mesh disks were cleaned with isopropanol and incubated in a 0.5% aqueous solution of PABT at 72ºC with shaking for 1 h followed by deprotection of thiol groups in the side chains of PABT with TCEP (12 mg/ml in 0.1 M acetate buffer at room temperature (RT) with shaking for 15 min). The samples were then extensively washed with degassed DDW and reacted under argon atmosphere with an 0.5% aqueous solution of PEI(PDT) at 28ºC with shaking for 1 h, washed and incubated with thiolated protein G (PrG-SH; 125 µg/ml in degassed PBS) at 28ºC with shaking for 1 h. The specimens were washed with PBS and reacted with 50 µg/ml of the anti-AAV2 antibody with shaking at RT for 45 min. The samples were then washed in PBS and incubated with AAV2 suspension (10^10^ VG/ml in PBS) at RT with mild shaking for 45 min to immobilize the vector particles on the metal surface.

### Cell culture

All primary cells were used in passages 3 to 7. RAEC and rat BOEC were grown in EGM-2 medium. All other cells were maintained in DMEM supplemented with 10% FBS (Gemini) and 1% antibiotic/antimycotic mixture (Gibco). When appropriate, the medium was switched to respective basal medium supplemented with lipoprotein-free FBS (Kalen Biomedical) to avoid the effects of HDL and apoA1 derived from serum on experimental endpoints.

### Transduction experiments

Rat BOEC, RAEC, RASMC and Raw264.7 cells were transduced with AAV2-apoA1(WT) and AAV2-apoA1(4WF) at MOI of 10^5^–5 × 10^5^ (10^10^–3.5 × 10^10^ VG/ml). The respective culture media were formulated using a lipoprotein-depleted FBS. The media were collected after 3 days of culture, and the concentration of human apoA1 in the conditioned media was assayed with ELISA. For immunofluorescence studies, RAEC and RASMC were transduced with AAV2-apoA1(WT) and AAV2-apoA1(4WF) as above. Three days post-transduction the cells were fixed in cold methanol and were stained with anti-apoA1 antibody (Abcam, ab52945), followed by either Alexa-488 or Alexa-549 labelled secondary antibodies. Properly stained untransduced RAEC and apoA1-transduced RASMC not exposed to the primary antibody served as controls.

### Cholesterol efflux experiments

HEK 293 cells were seeded in a 24-well plate. Upon reaching 75–85% confluence, the triplicate wells were transduced with AAV2-apoA1(WT), AAV2-apoA1(4WF) (both at MOI of 10^5^) or were left untransduced. Forty-eight hours after transduction the medium was changed for unsupplemented DMEM. After 24 h, the medium was collected and concentrated 20-fold using centrifugal concentration devices with a 3 kDa cut-off membrane. The concentration of human apoA1 in each preparation was then determined by human apoA1 ELISA (R&D Systems) per manufacturer instructions. ApoA1 concentration in the conditioned media originated from the AAV2-apoA1(WT)- and AAV2-apoA1(4WF)-transduced cells was then adjusted with unsupplemented DMEM to 10 µg/ml. Likewise, the media from untransduced cells was spiked with the commercial recombinant human apoA1 to 10 µg/ml concentration.

Murine Raw 264.7 macrophages in a 12-well plate format were treated with 0.5 µCi ^3^H-cholesterol (Perkin-Elmer) formulated in sterile culture media with the addition of 0.15 µM acetylated human LDL and incubated for 48 h to allow ample cholesterol uptake by macrophages. Twenty-four hours before testing, 8Br-cAMP was added to the medium at the final 0.15 mM concentration to activate the ABCA1 production. Immediately before starting the cholesterol efflux phase of the experiment, concentrated conditioned media from HEK-293 cells containing 10 µg/mL of apoA1(WT), 10 µg/mL apoA1(4WF), or 10 µg/ml of recombinant human apoA1 spiked to the media from untransduced cells were mixed with increasing concentrations of hypochlorous acid (0, 2, 8, and 16:1 molar ratio to apoA1). ApoA1 oxidation reaction was run in triplicate aliquots at 37 °C for 1 h. Raw 264.7 cells were then carefully washed with PBS and incubated with the differently oxidized HEK 293-conditioned DMEM in the cell culture incubator for 4 h. The media were then collected and centrifuged to exclude cell debris. Cells were washed with PBS, scraped, resuspended in PBS. 5 mL of Ecolite + scintillation fluid was added to each media or cell containing scintillation vial and vortexed. ^3^H count rates were then determined by using a scintillation counter (Beckman-Coulter LS6500). The ^3^H-cholesterol efflux was calculated as a percentage of ^3^H in the media over the total ^3^H-cholesterol content in both the media and cells.

### Proliferation assay

RASMC and rat BOEC grown to 70–80% confluence in T-75 flasks were transduced at MOI 10^5^ (10^10^ VG/ml) with AAV2-apoA1(WT), AAV2-apoA1(4WF), AAV2-eGFP or left untransduced. Four days after transduction, the cells were trypsinized, collected by centrifugation, mixed with FBS/DMSO, aliquoted and frozen. The single aliquots of non-transduced (NT), AAV2-eGFP-transduced, AAV2-apoA1(WT)-transduced, and AAV2-apoA1(4WF)-transduced cells of both types were then reseeded into the 96-well plates (N = 4 wells per group; 5 × 10^3^ cells/well across all experimental conditions). A group of wells in each plate was seeded at a higher density (10^5^ cells/well) to achieve immediate confluence. The cells were maintained in DMEM supplemented with 10% lipoprotein-free FBS (Kalen). 20 ng/ml of rat TNFα was added to RASMC cultures to emulate the atherosclerotic milieu. The relative cell number for each transduction type was determined at days 2 and 4 with WST-8 assay by normalizing the optical density values for each well of growing cells to that of 100% confluent reference wells. Cell growth kinetics were expressed as the percent of a monolayer confluency. At the completion of the WST-8 assay at day 4 post-seeding, the cells were fixed with 10% formalin and immunostained with rabbit anti-Ki67 antibody/goat anti-rabbit AlexaFluor 555-labelled antibody and counterstained with Hoechst 33,342.

### Migration assay

RASMC and rat BOEC pre-transduced *en masse* with AAV2 apoA1(WT), AAV2-apoA1(4WF), and frozen as detailed above for the proliferation assay, were seeded in a 48-well plate and cultured for 3–4 days in the respective media supplemented with 10% lipoprotein-depleted FBS until confluent. The cells were then starved for 36 h in media containing 0.5% lipoprotein-depleted FBS. A linear scratch injury was then inflicted to each well with a 200 µl pipette tip. The cells were washed with PBS to remove debris and imaged at 40 × magnification immediately after the scratch injury and 24 h after. The closure of the gap by inwardly migrating cells was quantitated using Image J (v1.53a).

### ROS assay

Rat aortic endothelial cells (RAEC) grown to 60–70% confluence in a 96-wells plate were either transduced with AAV2-apoA1(WT), AAV2-apoA1(4WF) at MOI of 5 × 10^5^ (3.5 × 10^10^ VG/ml) or left untransduced (N = 12 wells for each treatment). Seventy-two hours after transduction, the cells were washed with PBS and added DMEM supplemented with 10% lipoprotein-depleted FBS. Five hours after the medium change, half of the wells were treated with 40 ng/ml rat TNFα. Twenty-four hours after boosting ROS production with TNFα, the cells were washed with PBS and treated with 10 µm CM-H2DCFDA for 30 min, followed by fluorimetry at 485/538 nm. Additionally, ROS mitigation with apoA1(4WF) transduction (MOI of 5 × 10^5^; 3.5 × 10^10^ VG/ml) was assessed in tBHP-stimulated RAEC using CellROX Orange reagent (N = 5 wells per condition).

### Monocyte adhesion assay

Rat aortic endothelial cells (RAEC) grown to 60–70% confluence in a 96-wells plate were either transduced with AAV2-apoA1(WT), AAV2-apoA1(4WF) at MOI of 5 × 10^5^ or left untransduced (N = 4 wells for each condition). Seventy-two hours after transduction, the cells were washed with PBS, administered DMEM supplemented with 10% lipoprotein-depleted FBS and treated with 40 ng/ml rat TNFα for 24 h.

Monocytes were isolated from 10 ml of heparinized blood harvested from naïve male Sprague–Dawley rats by Ficoll-Paque gradient centrifugation with subsequent magnetic immunoseparation using a cocktail of anti-CD8, anti-CD5, anti-CD45RA, and anti-pan T cell antibodies^61^. Isolated monocytes were then fluorescently labeled with PKH-26 dye (Millipore-Sigma, St. Louis, MO, USA) as directed by a manufacturer. 5 × 10^4^ fluorescently-labeled monocytes were then added to each well with differently transduced, TNFα-activated rat endothelial cells. Following 30 min incubation in the cell culture incubator, monocyte adhesion was examined by fluorescence microscopy. The number of monocytes attached to the activated RAEC monolayers was derived from the 100 × magnification images of the central area of each well.

To eliminate the possibility that cell signaling events triggered by RAEC transduction with AAV2 vector decrease monocyte adhesion to the endothelial monolayer, in a separate experiment PKH-26 labeled rat monocytes were added to the wells of AAV2-Egfp—transduced and non-transduced RAEC stimulated with 40 ng/ml rat TNFα (N = 4 per condition).

### Scanning electron microscopy

The mesh disks formulated with 10^10^ VG/ml of AAV2-eGFP at the vector incubation step were washed in PBS and cacodylate buffer (pH 7.4) and fixed with 2% glutaraldehyde/cacodylate buffer. The samples were dehydrated with graded ethanol and hexamethyldisalazane, sputter-coated with gold/palladium alloy and imaged using a Quanta250 scanning electron microscope (FEI, Hillsboro, OR).

### Quantification of AAV2 load on stents and in the arterial wall

The extra AAV2-carrying stents not used in the animal experiments (N = 3), as well as stents (N = 3) harvested from the animal that died within 1 h of the stenting surgery, were carefully cut into 1–2 mm fragments. Arterial tissue segments (N = 3) underlying the stents harvested from the pig coronaries were separated from the metal struts and processed separately. Specifically, four fragments were excised from the central part of each arterial segment and pooled. The wet weight of the pooled specimens was ~ 30 mg. The tissue was minced using stainless steel beads and a Bullet Blender (both from Next Advance, Troy, NY). All specimens were then individually processed using QIAamp DNA minikit (Qiagen) to isolate the viral DNA. Calibration curve samples spanning 10^4^–10^10^ AAV2 genomes were prepared from the stock solution of the vector. An RT-PCR reaction was then carried out with AAV2-specific primers, thus providing direct quantification of viral genomes associated with each specimen. The fractions of the AAV2 load associated with the retrieved stents and the underlying arterial tissue were then calculated, assuming the viral load of undeployed stents as 100%. To verify completeness of viral DNA extraction from the stents, a second round of DNA extraction from the already processed samples was attempted in the preliminary experiments, yielding no DNA.

### AAV2-GFP stent reporter study

To study transduction of arterial tissue with AAV2-carrying stents in a pig model, 4 Yorkshire domestic pigs (22–28 kg) of both genders received AAV2-eGFP stents in the left anterior descending (LAD) and the circumflex (Cx) coronary arteries. The animals were euthanized 7 days after the surgery, and the harvested stented arteries (N = 8) were snap-frozen in liquid N_2_, pulverized under liquid N_2_, and the tissue powder was suspended in 500 µl of T-Per buffer (Thermo Scientific) supplemented with protease inhibitors (Roche), incubated on ice for 30 min, and centrifuged at 10,000 G for 10 min. Protein concentration in the supernatant of each sample was determined by the BCA assay. Fifty µg protein samples were resolved on a 4–12% NuPAGE™ Bis–Tris polyacrylamide gel, blotted to a nitrocellulose membrane, blocked with 5% dry milk/PBS and consecutively reacted with anti-eGFP antibody (Rockland, 1:7000 dilution) and anti-β tubulin antibody (GeneTex, 1:5000 dilution), peroxidase-conjugated goat anti-rabbit antibody (Santa Cruz; 1:2000 dilution) and SuperSignal™ Pico Plus luminescent substrate (Thermo Scientific). The signal was detected using a Luminescence detection station (IVIS Spectrum) and analyzed with Image J software (v1.53a).

### Hypercholesterolemic/diabetic pig model and pig stenting experiments

All animal experiments were pre-approved by the Institutional Animal Care and Use Committee of the University of Pennsylvania, carried out in accordance with federal regulations and reported in accordance with ARRIVE guidelines. To test the therapeutic effectiveness of stents formulated with AAV2-apoA1(4WF) vector immobilized on the bare metal stent struts, a hypercholesterolemic/diabetic pig model^62^ was used. Before instituting model-related pharmacological and dietary interventions, all animals were screened for the presence of preformed anti-AAV2 antibodies, and the animals tested positive were either excluded from the study or were ascribed to the group treated with BMS (Supplemental Fig. 6). Totally eleven 10–12 week-old female Yorkshire domestic pigs (22–28 kg) were made diabetic by an intravenous injection of Streptozotocin (125 mg/kg). After verification of persistent hyperglycemia (> 250 mg/dl) the animals were administered a hypercholesterolemic diet (0.5% cholesterol, 5% lard, 1.5% sodium cholate) for 24 weeks. Total blood cholesterol and blood glucose levels were monitored throughout the study. If blood glucose exceeded 400 mg/dl, insulin was administered as needed.

All study animals underwent cardiac catheterization with coronary angiography. Bare metal stents, AAV2-eGFP stents, and AAV2-apoA1(4WF) stents (all 18-mm length, mounted onto 3 mm or 3.5 mm balloon catheters) were implanted in proximal and/or distal locations of each animal’s left anterior descending (LAD) and the circumflex (Cx) coronary arteries (2–4 stents per animal). An inflation pressure of 10–14 atm was applied to deploy stents to achieve a 1.1–1.2 stent/artery diameter ratio. Two animals died because of ventricular fibrillation within 1 h of stenting (Supplemental Fig. 6). The harvested arteries of one of the deceased animals were used to quantify the vector load on the stents and the vector loss during deployment and the initial phase of the release. All survived animals were euthanized 4 weeks after stent deployment by IV injection of KCl (125 mg/kg). The hearts were harvested. The stented arterial segments were excised preserving the 2–3 mm non-stented flaps of arterial tissue on both sides and flushed with heparinized saline. The stent-free overhangs were then dissected and snap-frozen in liquid nitrogen for RT-PCR studies. The stented portions of the arteries were fixed in 10% buffered formalin, methyl methacrylate-embedded, sectioned, deplastified and stained according to the Verhoeff-van Giesson method. Five sections cut 3 mm apart from each other through the entire length of the stented segment were stained and analyzed. Ends of the stented segments were excluded. Digital images of the stained arterial sections were captured at 20 × magnification, and the areas of the lumen, internal, and external elastic laminas were measured to derive the extent of restenosis, expressed as a neointima-to-media area ratio, % of luminal stenosis and neointimal thickness. These restenosis indices from 5 individual sections were averaged and the mean values were used for comparison between the different treatment groups.

### Human apoA1(4WF) expression in the GDS-treated porcine arteries

Snap-frozen arterial samples adjacent to stents were stored at −80 °C until processing. The samples were homogenized with steel beads using a Bullet Blender (Next Advance, Troy, NY). RNA was extracted using a RNeasy Fibrous Tissue Mini Kit (Qiagen, Germantown, MD). Five hundred µg of isolated RNA were converted into cDNA with TaqMan reverse transcription reagents (Applied Biosystems, Waltham, MA). RT-PCR reaction was carried out in a 7500 fast Real-Time PCR engine using the following primer sequences for amplification of apoA1(4WF) cDNA: (fwd: 5’-TTT-GAT-CGA-GTG-AAG-GAC-CTG-3’ and rvs: 5’-GGT-TAT-CAA-AGA-ACT-CCT-GGG-T-3’). A ddCt algorithm was applied for the interpretation of RT-PCR results. Porcine β2 microglobulin housekeeping gene amplification with the respective primers (fwd: 5’-CGC-CCC-AGA-TTG-AAA-TTG-ATT-TGC-3’ and rvs: 5’-GCT-ATA-CTG-ATC-CAC-AGC-GTT-AGG-3’) was used for the normalization, and the arterial tissue harvested adjacent to the BMS was used as a reference sample.

### AAV2-neutralizing antibodies titering

To determine the prevalence of AAV2 neutralizing antibodies in the serum of experimental animals, blood sampled at the beginning of the study, 1 week before the intervention, and at the sacrifice was allowed to clot and centrifuged at 1500G for 15 min. Obtained sera were stored at −80 °C prior to analysis. HEK-293 cells grown in the 96-well plates to 80% confluence were transduced with AAV2-eGFP at MOI of 10^5^ in the presence of 1:20 diluted serum samples or without the addition of the sera. The ensuing transgene expression was determined by fluorimetry at 485/538 nm 3 days after transduction. 50% or higher inhibition of transgene expression manifested as reduction of eGFP fluorescence intensity, denoted presence of neutralizing antibodies in the serum. The animals that exhibited the presence of neutralizing antibodies at the beginning of the study were excluded from the study or slated to the BMS treatment group.

### Statistical methodology

Data are presented as means ± SD, unless specified otherwise. Differences between the groups were analyzed by ANOVA followed by a post-hoc Tukey’s test, and were termed statistically significant at p < 0.05.

## Supplementary Information

Supplementary Information 1.

Supplementary Information 2.

## Abbreviations

apoA1: Apolipoprotein A1
HDL: High density lipoproteins
BOEC: Blood outgrowth endothelial cells
RAEC: Rat aortic endothelial cells
RASMC: Rat aortic smooth muscle cells
GDS: Gene delivery stents
ISR: In-stent restenosis
NA: Neoatherosclerosis
LST: Late stent thrombosis
DES: Drug eluting stents
CVD: Cardiovascular diseases
MPO: Myeloperoxidase
PABT: Polyallylamine bisphosphonate with latent thiol groups
PEI(PDT): Polyethyleneimine with installed pyridyldithio groups
TCEP: Tris(2-carboxyethyl)phosphine hydrochloride
DDW: Double distilled water
RT: Room temperature
MOI: Multiplicity of infection
VG: Viral genomes
tBHP: Tert-butyl hydroperoxide
