# Supplementary material for: Stent-based delivery of AAV2 vectors encoding oxidation-resistant apoA1
Source: Sci Rep. 2022 Mar 31;12:5464. doi: 10.1038/s41598-022-09524-y (PMC8971450; doi:10.1038/s41598-022-09524-y)
Supplement: Supplementary file 1 — Supplementary Information 1. [file 41598_2022_9524_MOESM1_ESM.pptx]

## Slide 1
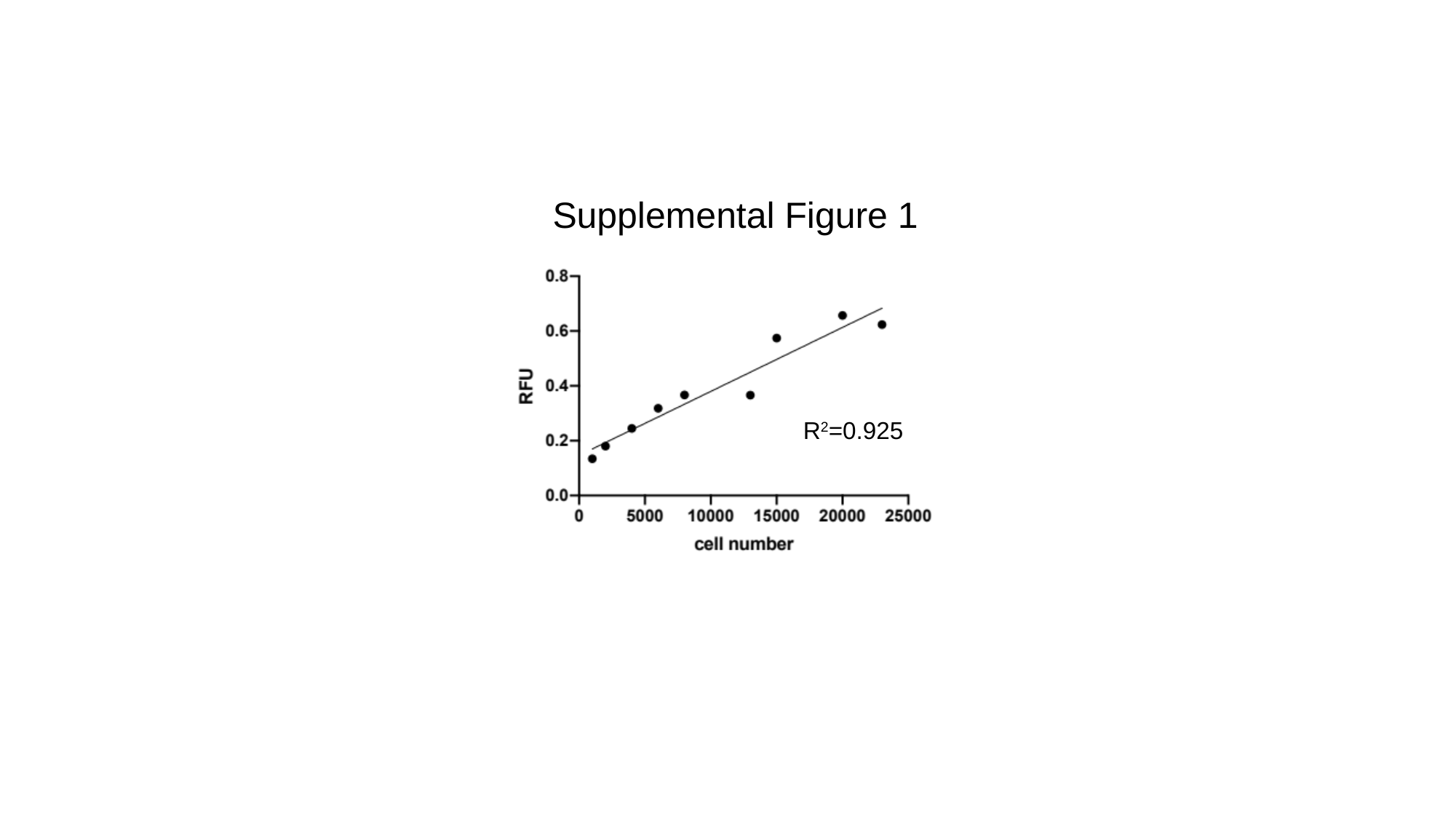

Supplemental Figure 1
R2=0.925

## Slide 2
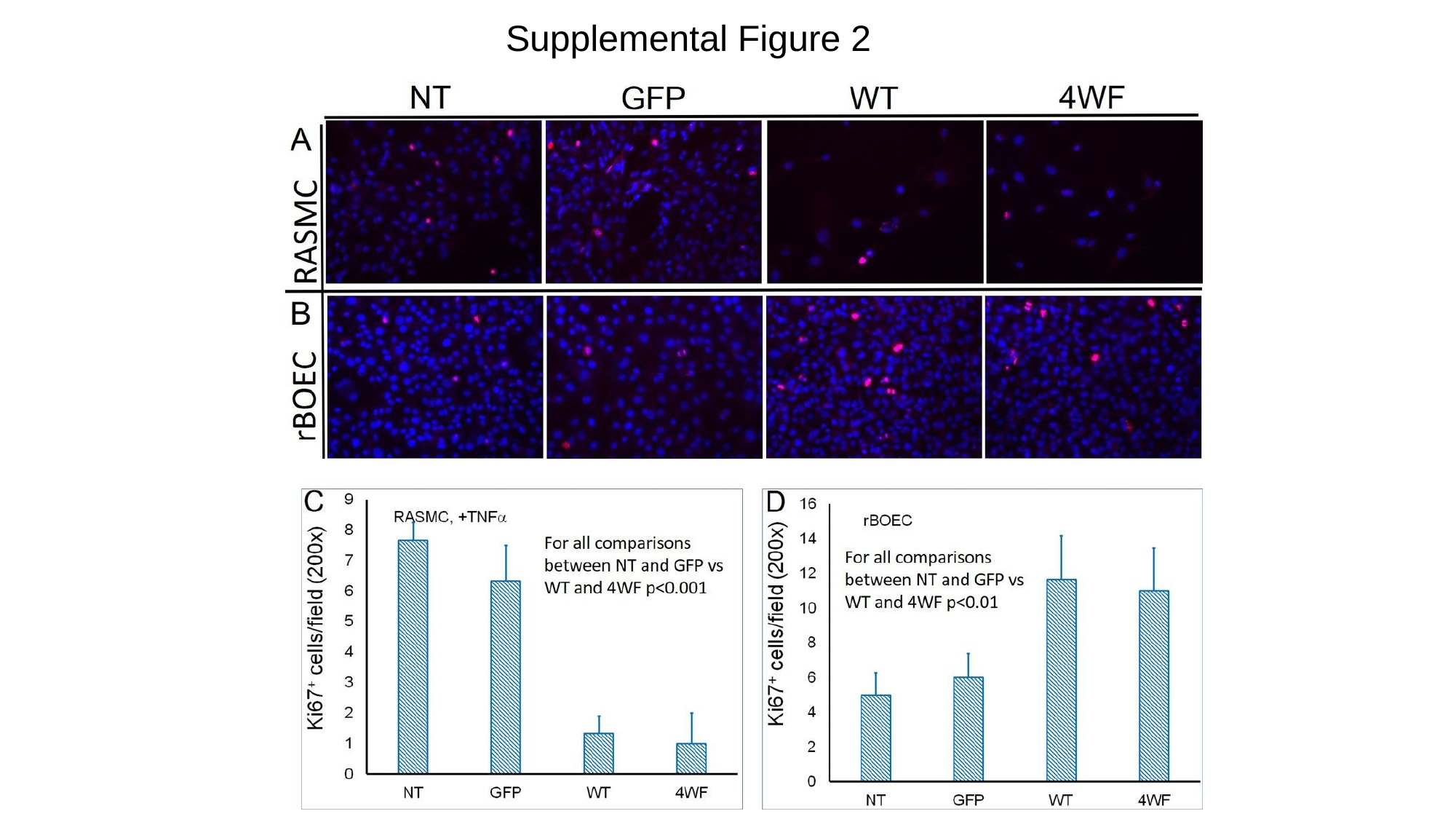

Supplemental Figure 2

## Slide 3
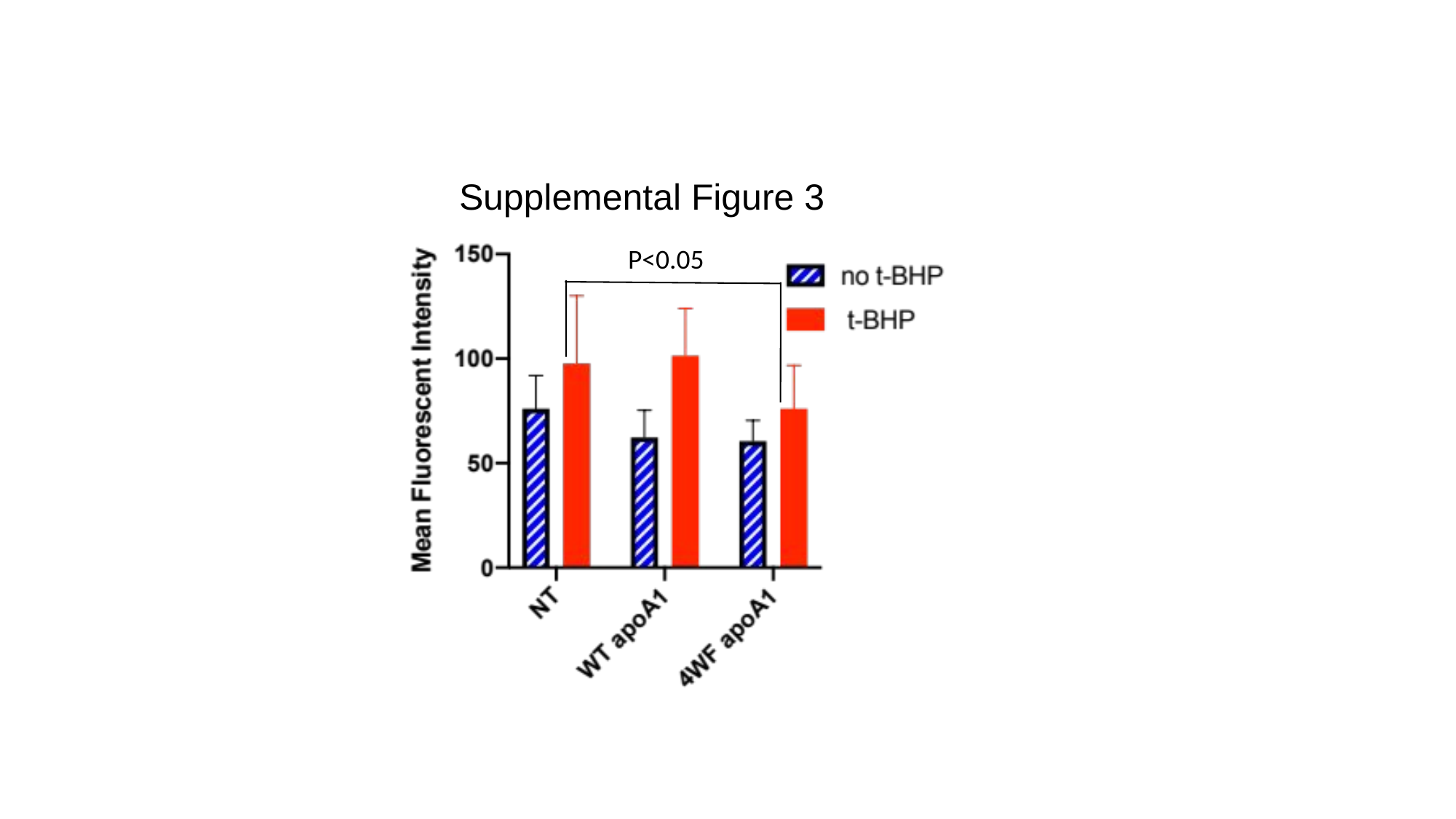

Supplemental Figure 3
P<0.05

## Slide 4
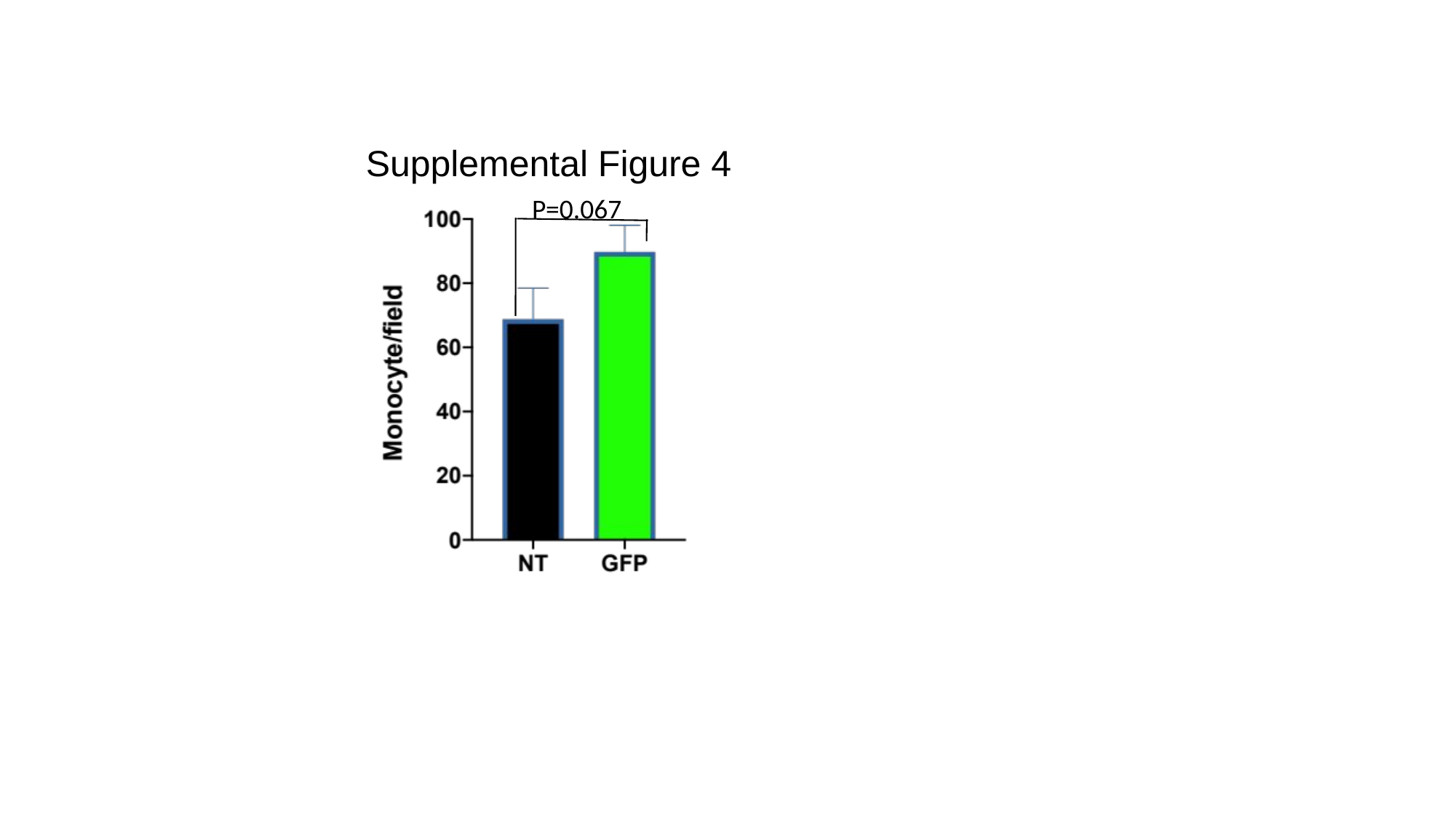

Supplemental Figure 4
P=0.067

## Slide 5
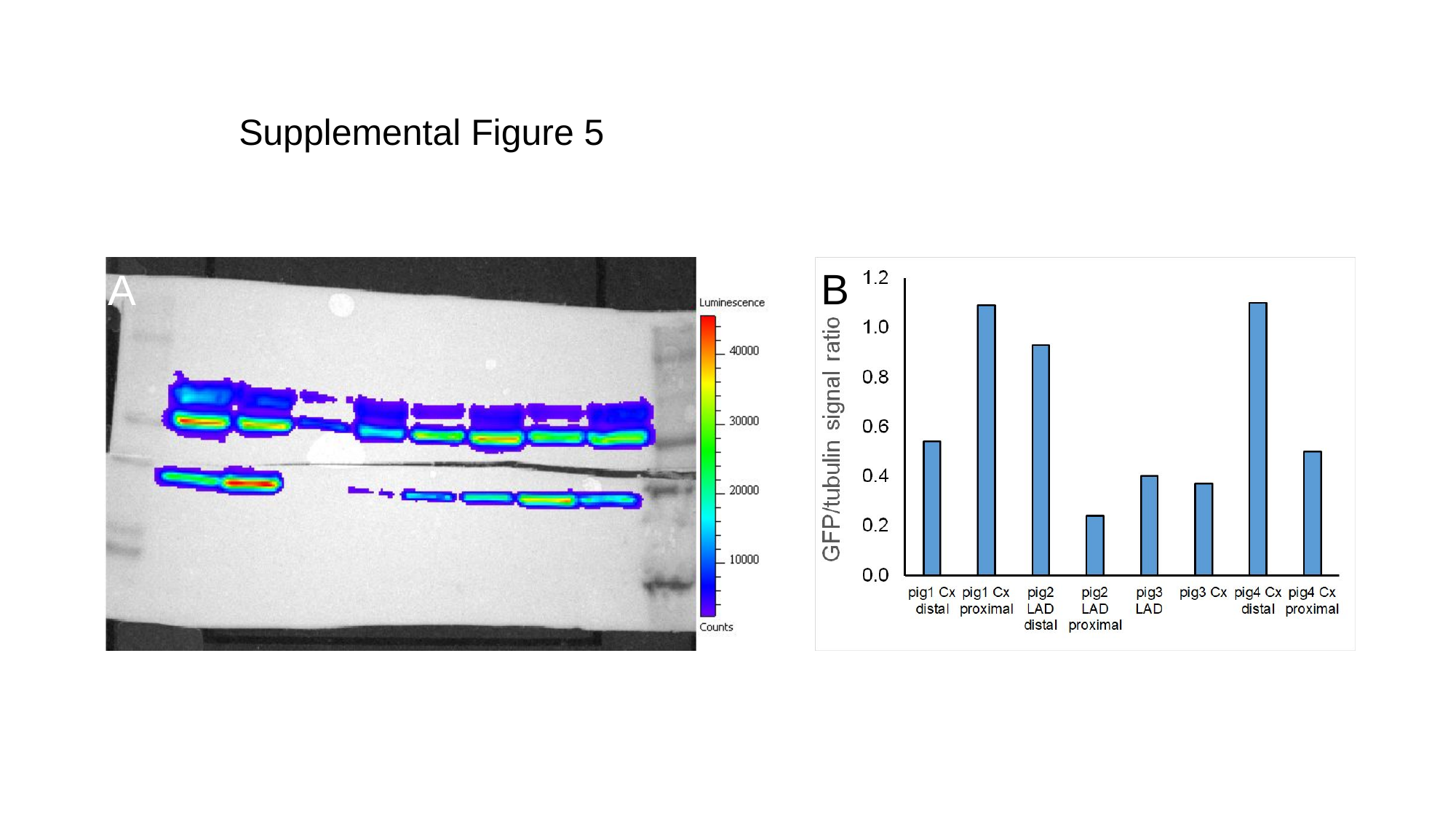

Supplemental Figure 5
B
A

## Slide 6
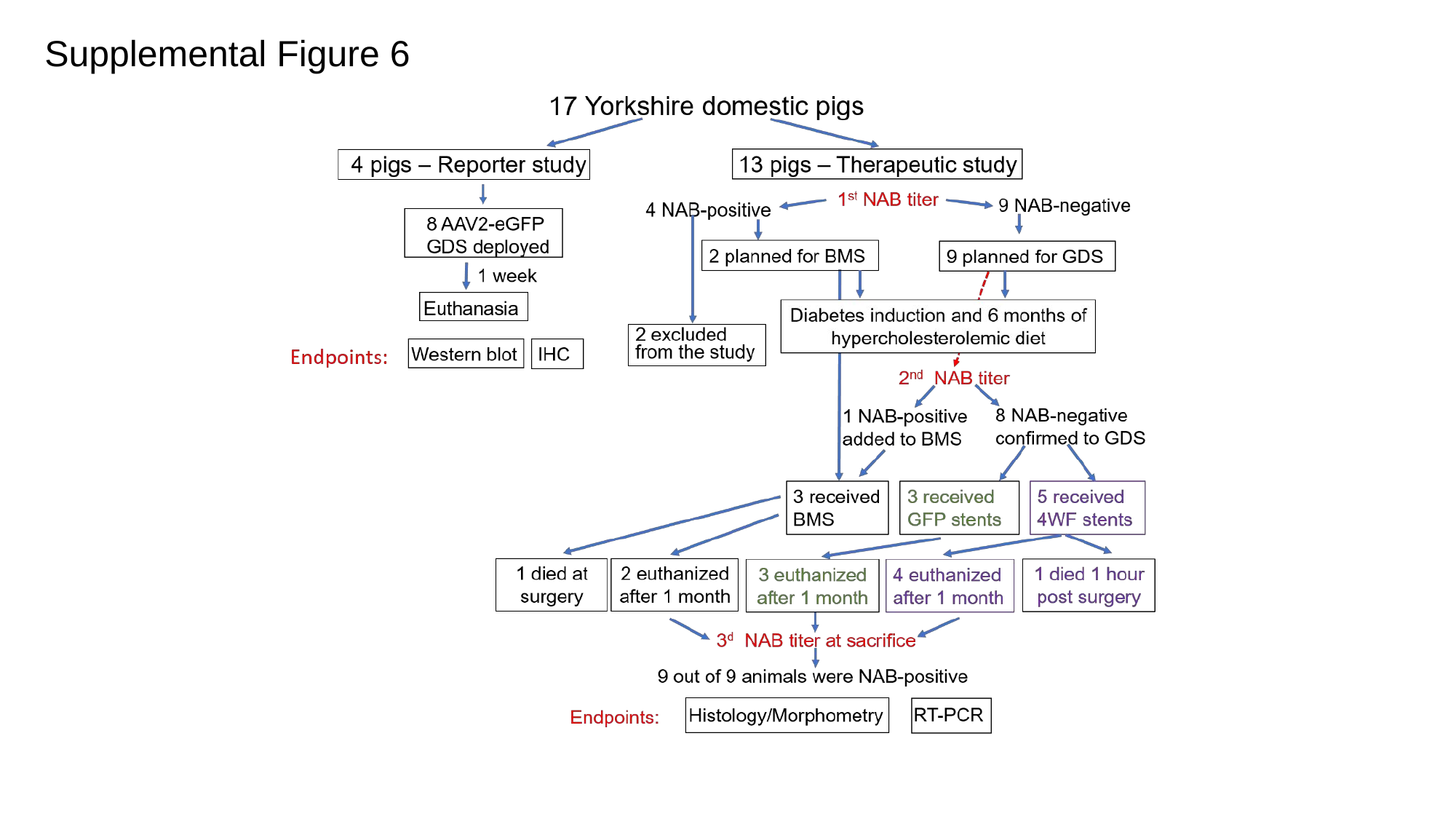

Supplemental Figure 6
