# Supplementary material for: Stent-based delivery of AAV2 vectors encoding oxidation-resistant apoA1
Source: Sci Rep. 2022 Mar 31;12:5464. doi: 10.1038/s41598-022-09524-y (PMC8971450; doi:10.1038/s41598-022-09524-y)
Supplement: Supplementary file 2 — Supplementary Information 2. [file 41598_2022_9524_MOESM2_ESM.docx]

SUPPLEMENTARY INFORMATION

**Supplemental Figure 1**. **Validation of WST-8 assay against direct cell counting.**

Rat BOEC were seeded in escalating numbers into the individual wells of a 96-well plate (n=4 for each seeding density). Twelve hours after seeding, the relative cell amounts in all wells were quantified using the WST-8 reagents, followed by trypsinization and manual cell counting using a hemocytometer.

**Supplemental Figure 2**. **Effects of AAV2 transduction with apoA1 variants on rat SMC and rat BOEC proliferation.**

Ki67 immunocytochemistry was used to compare proliferation in non-transduced (NT), and eGFP-, apoA1-WT- and apoA1-4WF-transduced RASMC (A, C) and rat BOEC (B, D) after 4 day culture.

**Supplemental Figure 3. Effects of apoA1(4WF) expression on basal and tBHP-stimulated ROS production in RAEC.**

Non-transduced RAEC and RAEC transduced with AAV2-apoA1(4WF) were loaded with CellROX Orange ROS indicator either with (solid bars) or without (striped bars) stimulation with tert-butyl hydroxyperoxide (t-BHP). The representative fluorescence images (rhodamine filter set) were saved as TIFF files, and the mean fluorescent intensity of 10 cells in each image was determined using the Photoshop™ histogram tool.

**Supplemental Figure 4. Effects of AAV2 transduction with inflammation neutral transgene on monocyte attachment to RAEC monolayer.**

Attachment of PKH26-labeled rat monocytes to TNFα-stimulated eGFP-transduced and non-transduced RAEC (N=4 per group).

**Supplemental Figure 5. Unretouched Western blot image associated with the main text Fig 5 and tubulin-normalized eGFP expression.**

(A) An IVIS Lumina-generated Western blot image of PAGE-resolved, PVDF-transferred proteins from AAV2-GFP stent-treated porcine arteries.

After transfer, the PDVF membrane was cut between the 31 and 38 kDa marks of the transferred molecular weight ladder. This was done to allow the separate use of 2 antibodies targeting the proteins with the molecular weight of 28 kDa (eGFP) and 55 kDa (b-tubulin). The original blot was not cropped or otherwise tempered. Top and bottom margins of the membrane are clearly visible. Molecular weight markers appear left and right. The image was taken as a combined luminescence/photographic image using an IVUS Spectrum bioluminescence station and Living Image software (version 4.2). The original Living Image file is available and can be provided upon request. To obtain the Western blot image labeled Figure 5 in the main text, a black and white version of the original blot was generated. The image was rotated 4ﹾ counterclockwise and the molecular weight ladder was annotated. (B) Densitometry-based eGFP/beta-tubulin ratio in the individual stented vessels.

**Supplemental Figure 6. Flow diagram of animal experiments.**
